# Effects of tDCS of Dorsolateral Prefrontal Cortex on Dual-Task Performance Involving Manual Dexterity and Cognitive Task in Healthy Older Adults

**DOI:** 10.3389/fnagi.2019.00144

**Published:** 2019-06-18

**Authors:** Milos R. Ljubisavljevic, Joji Oommen, Sasa Filipovic, Jovana Bjekic, Miklos Szolics, Nico Nagelkerke

**Affiliations:** ^1^Department of Physiology, College of Medicine and Health Sciences (CMHS), UAE University, Al Ain, United Arab Emirates; ^2^Department of Neuroscience, Institute for Medical Research, University of Belgrade, Belgrade, Serbia; ^3^Department of Internal Medicine, Neurology Section, Tawam Hospital, Al Ain, United Arab Emirates; ^4^Institute of Public Health, College of Medicine and Health Sciences (CMHS), UAE University, Al Ain, United Arab Emirates

**Keywords:** tDCS, DLPFC, aging, motor dexterity task, cognitive task, dual-tasking

## Abstract

Healthy aging limits the activities of daily living and personal independence. Furthermore, cognitive-motor interference in dual-task (e.g., walking while talking) appears to be more pronounced in the elderly. Transcranial direct current stimulation (tDCS), a form of the non-invasive brain stimulation technique, is known to modify cortical excitability and has been investigated as a tool for enhancing motor and cognitive performance in health and disease. The present study examined whether tDCS targeting the dorsolateral prefrontal cortex (DLPFC) could improve dual-task performance in healthy older adults. The effects of tDCS, among other factors, depend on stimulation polarity (anodal vs. cathodal), electrode setup (unilateral vs. bilateral) and the time of application (off-line vs. on-line). We therefore explored the effects of unilateral and simultaneous bilateral tDCS (anodal and cathodal) of left DLPFC while performing (on-line) the Grooved Pegboard Test (GPT) and Serial Seven Subtraction Test (SSST) alone or together (dual-tasking). The number of pegs and the number of correct subtractions were recorded before, during and 30 min after tDCS. The dual-task performance was measured as the percent change from single- to the dual-task condition (dual-task cost DTC). Only bilateral, anode left tDCS, induced a significant increase in subtracted numbers while dual-tasking, i.e., it reduced the DTC of manual dexterity (GPT) to a cognitive task. Significant changes 30 min after the stimulation were only present after bilateral anode right (BAR) tDCS on GPT dual-task costs. These findings suggest that anodal tDCS applied on-line interacts with a dual-task performance involving demanding cognitive and manual dexterity tasks. The results support the potential use of non-invasive brain stimulation for improvement of cognitive functioning in daily activities in older individuals.

## Highlights

– Bilateral tDCS of the dorsolateral prefrontal cortex enhances dual-task performance in older subjects.
– Anode left-cathode right tDCS montage appears more effective than the opposite bilateral stimulation montage.
– Effects are more pronounced during stimulation than after stimulation.
– Effects are more pronounced on demanding cognitive task than on manual dexterity tasks.
– Results argue for further exploring the potential use of tDCS for cognitive enhancement in older subjects.

## Introduction

It is widely recognized that in normal aging brain undergoes complex structural and functional changes, giving rise to age-related deterioration of cognitive, perceptual, and motor abilities, affecting activities of daily living, independence, and overall quality of life (Craik and Bialystok, 2006). Cognitive aging affects multiple domains including executive functions and memory while age-related motor deficits are also pervasive including deterioration of control and execution of movements (Lord et al., 2018). Age-related cognitive and motor deficits are also believed to be related to more pronounced deterioration of the ability to perform two tasks simultaneously (multitasking cost) by older adults, compared to younger individuals (Kearney et al., 2013; Schoene et al., 2014).

Transcranial direct current stimulation (tDCS) is a non-invasive brain stimulation technique in which electrical current passed through the skull induces changes in membrane potential of cortical neurons (Nitsche and Paulus, 2000; Nitsche et al., 2003) thus modulating cortical excitability (Hummel and Cohen, 2005). Depending on the direction of the current flow between the electrodes it either increases (anodal polarization) or decreases (cathodal polarization) the activity of the underlying neurons (Nitsche et al., 2008). It is believed that these effects on the excitability are related to transient changes in the synaptic efficacy of different neurotransmitter systems (Nitsche et al., 2008). The literature on tDCS for improvement of cognitive and motor performance in various tasks and age groups is vast (Bennabi et al., 2014; Parasuraman and McKinley, 2014; Ammann et al., 2016). Nevertheless, only a few studies explored the use of tDCS to improve dual-task performance. Single tDCS of the posterior lateral prefrontal cortex (pLPFC) reduced the cost of performing a secondary simultaneous cognitive task on gait and postural control in healthy young adults (Zhou et al., 2014) and reduced the cost of responding to simultaneous visual and auditory stimuli (dual-task; Filmer et al., 2013). Along the same lines, tDCS of the left PFC altered dual-task gait and cognitive task performance in a polarity-dependent manner (Wrightson et al., 2015), and reduced dual-task costs to standing or walking while performing serial subtractions, without affecting the performance of each task (Manor et al., 2016). In older adults, a single tDCS session targeting PFC increased standing postural sway complexity with concurrent non-postural cognitive tasks (Zhou et al., 2015). All of these studies examined unilateral tDCS, where an active electrode is placed over the region of interest over the left or the right hemisphere and the return electrode is placed over the contralateral supraorbital area or an extracephalic area (Nasseri et al., 2015). Unlike in unilateral stimulation in bilateral tDCS anodal and cathodal stimulation are simultaneously applied over homologous cortical brain regions aiming to enhance the excitability in one region, while at the same time reducing the excitability in the opposite hemisphere (To et al., 2018). In older adults, it has been suggested that bilateral tDCS produces modulation of complex networks involving interhemispheric interactions, of primary target areas, as well as broader cortical networks including the dorsal posterior cingulate cortex (Lindenberg et al., 2013). Furthermore, most of the studies that examined the effects of tDCS on dual-task performance tested them after the stimulation (off-line), rather than during tDCS. Applying tDCS during task performance (on-line design) aims to facilitate motor performance by enhancing the activity of task-related networks and strengthening of relevant synaptic connections (e.g. Oldrati et al., 2018).

Thus, this study aims to examine whether bilateral, rather than unilateral, tDCS of the dorsolateral PFC (DLPFC), applied while performing dual-task, is more effective in improving dual-task performance in older adults. We used a modification of a novel dual-task method to assess cognitive impairment in older adults involving upper-extremity dual-task function (Toosizadeh et al., 2016).

## Materials and Methods

### Subjects

Twenty-six healthy older subjects volunteered to participate, out of which 22 fulfilled all conditions, completed the study and were included in the analysis (mean ± SD, age 62.6 ± 3.2 year; range 57–71; 6 women, 16 men). Subjects were recruited by open email invitation and by “word of mouth” mostly amongst UAE University academic staff. All subjects were highly educated with a minimum of BSc degree. They were all right-handed according to the Edinburgh inventory (Oldfield, 1971). All subjects gave written informed consent to participate in the experiment in accordance with the Declaration of Helsinki. The Al Ain Medical District Human Research Ethics Committee approved the study (Protocol No. 14/57). All participants were healthy as confirmed by a screening medical and neurological examination and were not using any centrally-acting medication or had any other condition that may have influenced their performance. To further ensure that the subjects meet standard cognitive “normality” criteria, before the start of the study, they were tested using Mini-Mental State Examination (MMSE; mean ± SD: 29.1 ± 1).

### tDCS

tDCS was delivered using a battery-driven electrical stimulator (Soterix Medical, New York, NY, USA), set to deliver 1.5 mA current, through a pair of saline-soaked 35 cm^2^ (pad size 5 × 7 cm) surface sponge electrodes placed on the scalp, giving the current density of 0.042 mA/cm^2^. This amount of stimulation is safe (Poreisz et al., 2007; Antal et al., 2017) and has been shown to induce acute changes in cortical excitability (Nitsche and Paulus, 2000). The duration of stimulation was 30 min, with a ramp-up, -down each lasting 30 s. The SHAM stimulation also involved 30 s of ramp-up, -down current, at the beginning and end of stimulation session, respectively (Soterix Medical sham waveform), and no current during the session, which is accepted routine for ineffective stimulation (Gandiga et al., 2006). Ramp-up and -down period further helped that the subjects remained “blind” for the type of stimulation they received ensuring a sham control effect (Nitsche et al., 2008). Apart from tingling and itching at the beginning of the stimulation, the subjects did not report any significant discomfort or unpleasant sensations (e.g., pain). Also, when asked whether they experienced any adverse effects after the stimulation session the subjects reported no adverse effects.

### Tasks

A manual dexterity Grooved Pegboard Test (GPT) was given using standard Lafayette Instrument Company (Lafayette, IN, USA) 32025 board. The Grooved Pegboard is a manipulative dexterity test in which pegs must be rotated to match the hole with randomly positioned slots before they can be inserted. The test measures performance speed in a fine motor task, which requires more complex sensory-motor coordination. The performance on GPT is insensitive to handedness but sensitive to sex (Bornstein, 1986; Bryden and Roy, 2005). The test was performed according to the standardized method. The subjects were asked to insert the pegs into holes on the board one by one with the right hand, as quickly as possible, according to a standard sequence. The number of correctly inserted pegs within 30 s was counted.

The second task was a complex cognitive task of Serial Seven Subtractions (SSST). The starting number was randomly generated in the range between 290 and 330. The subjects were instructed to count backward every 7th number as quickly as possible within 30 s. The numbers were recorded on the paper, and the number of correct responses was counted (if seven was subtracted from the previous number correctly, it was regarded as a correct calculation).

In dual-task, the selected initial number was shown when the pegboard test was started, and the subjects performed both tasks simultaneously as quickly as possible within 30 s. Subjects were asked not to prioritize either of the two tasks (Montero-Odasso et al., 2012). The difference in the performance between single and dual tasks (i.e., dual-task cost-DTC) was calculated as the percent change in each variable from single- to dual-task condition (Hausdorff et al., 2008; Ullmann and Williams, 2011) according to the following formula: Change in dual tasks = (number in single task − number in dual tasks)/number in single task, so that negative values indicate better performance during dual- than single-task.

Finally, to account for general effects of tDCS on motor performance a simple reaction time (SRT) was examined using Psychology Experiment Building Language (PEBL; Mueller and Piper, 2014) freeware program working on a local computer. Subjects were asked to press a single button on a keyboard using the chosen digit of their right hand as quickly as possible in response to the appearance of visual patch stimulus presented on the computer screen. In total, 60 (3 × 20) stimuli were presented with an ISI of 0.5–3.5 s. All tests were performed on the same computer, with no changes in screen luminescence between sessions.

### Study Design and Experimental Conditions

**Figure 1:**
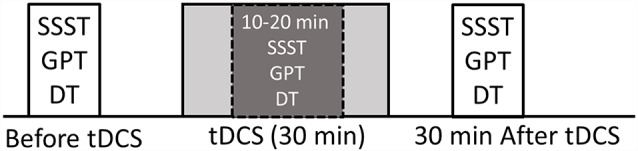
Experimental design. Serial Seven Subtraction Task (SSST; count backward every 7th number as quickly as possible for 30 s). Grooved Pegboard Task (GPT; insert pegs into holes on the board one by one with the right hand, as quickly as possible for 30 s). Dual-Task (DT; perform both SSST and GPT for 30 s). All tasks (every single-task and dual-task) were performed before, during and 30 min after transcranial direct current stimulation (tDCS). Tasks performed during tDCS were performed 10 min after the onset of stimulation.

We employed a sham-controlled, double-blind, randomized, repeated measures design, i.e., subjects and examiners scoring tasks were not aware of the tDCS condition, while the researcher administering tDCS was not involved in the assessment analysis. The blinding procedures were adequate since participant guesses of tDCS condition were not better than random (Fisher’s exact test *p* = 0.37). The order of the tDCS sessions was randomized between and within subjects. Sessions were separated by at least 2 weeks to minimize carryover effects. Unilateral tDCS was performed with the anode placed over the left or right DLPFC (i.e., F3 or F4, respectively, located using standard 10–20 EEG electrode position nomenclature) and the cathode placed over the contralateral supraorbital region, serving as a return electrode (Boggio et al., 2008). This montage is thought to induce facilitation of activity within the left PFC (under the anode; Wagner et al., 2007; Javadi and Walsh, 2012) and has been shown to enhance numerous cognitive functions acutely. Bilateral tDCS was performed with electrodes placed over left (F3) and right (F4) DLPFC (i.e., an anodal electrode over right, a cathodal electrode over left DLPFC and vice versa). For the sham condition, participants were randomly assigned to receive the sham stimulation using either the bilateral or unilateral montage. Therefore, all participants were tested on five separate occasions corresponding to following stimulation montages (sessions): unilateral anode left (UAL), unilateral anode right (UAR), bilateral anode left (BAL), bilateral anode right (BAR) and sham (Sham) stimulation. The experiments were conducted at the same time of the day (between 9 and 12 a.m.) to control for potential circadian effects (Sale et al., 2007). During each session, subjects were asked to perform each task (GPT, SSST, dual-task, and SRT) once before (PRE), once during (DUR) and once after (POST) tDCS (see Figure 1). Tasks were assigned randomly, between and within sessions. During tDCS tasks were performed between a 10th and 20th minute of stimulation according to the subject’s individual pace preference (Figure 1).

### Statistical Analysis

To test the effect of tDCS on SRT, we used two-way repeated measure analysis of variance (ANOVA). To compare single- vs. dual-task performance for each outcome separately in each session a paired-samples *t*-test was used. To study the effects of montage on performance we carried out a linear mixed effect (ANOVA, MIXED model using REML), with subject as random effect (random intercepts) of the relationship between performance on each task alone, during dual-task, and dual-task costs, and tDCS montage for each of the three time points (before, during and after tDCS) separately. Here, the montage was the fixed effect, and all “active” montages were compared to Sham as their reference. The significance level was set to 0.05 for all analyses. Both *p*-values for individual effects as well as *F*-tests for main (overall) effects are reported. *P*-values are all reported exactly (up to 3 decimals) so that readers can adjust for multiple comparisons using Bonferroni corrections where considered appropriate. SPSS 18.0 software (SPSS Inc., Chicago, IL, USA) was used for statistical analysis.

## Results

### Single-Task Performance and Simple Reaction Time (SRT)

Changes in the raw performance on single SSST and GPT tasks and the SRT across all experimental sessions are shown in Table 1 There was no main effect of stimulation montage on SRT (*F*_(4,84)_ = 1.416, *p* = 0.236) before (PRE), during (DUR; *F*_(4,84)_ = 1.088, *p* = 0.368) and after tDCS (POST; *F*_(4,84)_ = 1.032, *p* = 0.395) suggesting that tDCS did not influence SRT irrespective of the arrangement (montage) of DLPFC stimulation.

**Table 1:** Single-task performance: number of pegs inserted [Grooved Pegboard Test (GPT)] and number of correct subtractions [Serial Seven Subtraction Task (SSST)] within 30 s before (PRE), during (DUR) and 30 min after (POST), unilateral (Ul), bilateral (Bl) and sham transcranial direct current stimulation (tDCS) of left (AL) and right (AR) dorsolateral prefrontal cortex (DLPFC). Simple reaction time (SRT), before, during and after tDCS in all five montages.

	BAL	BAR	UAL	UAR	Sham
SSSTPRE	10.18 ± 2.3	10.04 ± 2.5	10.36 ± 2.6	10.22 ± 2.2	10.18 ± 2.4
SSSTDUR	10.31 ± 2.2	10.04 ± 2.1	10.27 ± 2	10.18 ± 2.3	10.4 ± 2.1
SSSTPOST	10.72 ± 2.4	10.59 ± 2.4	10.54 ± 2.6	10.63 ± 2.5	10.72 ± 2.7
GPTPRE	14.45 ± 2.1	14.72 ± 1.4	14.18 ± 2	14.22 ± 1.6	14.31 ± 1.8
GPTDUR	14.77 ± 1.9	14.31 ± 1.6	14 ± 1.8	14.13 ± 2.1	14.13 ± 2.1
GPTPOST	14.04 ± 1.9	14.27 ± 1.9	14.54 ± 1.7	14.4 ± 1.9	13.86 ± 2.1
SRTPRE	293.36 ± 41.1	300.59 ± 39.8	304.86 ± 37.3	301.4 ± 43.5	296.59 ± 46.8
SRTDUR	296.36 ± 40.9	306 ± 40.3	302.95 ± 39.5	296.31 ± 43.2	302.81 ± 41.9
SRTPOST	303.04 ± 44.6	299.86 ± 44.1	305.13 ± 42.4	295.45 ± 43	297.9 ± 37.5

*Simple reaction time (SRT), before, during and after tDCS in all five montages. Data are expressed as mean ± SD*.

The performance on both single-tasks was similar in all sessions before, during and after tDCS (Table 1). Furthermore, subjects’ performances on GPT were in line with the normative data for their ages. The mixed model examining the effects of the stimulation montage on single-task performance for each task separately showed no significant stimulation specific effects pre, during or after tDCS, as *p* > 0.05.

### Dual-Task Performance and Dual-Task Costs

**Figure 2:**
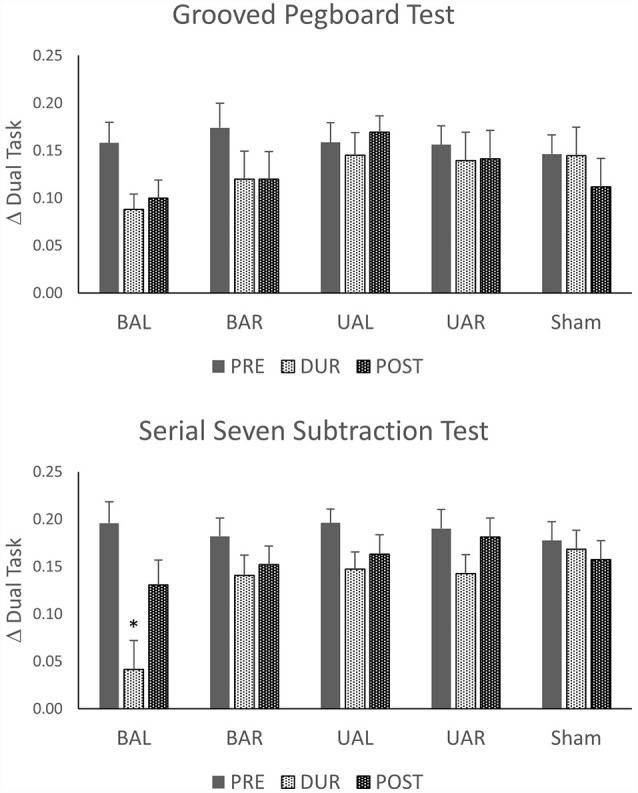
Changes from single- to dual-task condition calculated as change in dual task (Δ) = (number in the single task − number in dual tasks)/number in a single task. Stimulation tDCS montages: unilateral (Ul), bilateral (Bl), sham stimulation of dorsolateral prefrontal cortex (DLPFC) with anode left (AL) and anode right (AR). *p < 0.05.

Table 2 and Figure 2 summarizes the subject’s raw performance on dual-task and dual-task costs for all montages. Both SSST and GTP dual-task performance showed expected changes compared to single-task performance. On average the number of correct subtractions and the number of pegs inserted in 30 s decreased in dual-task compared to a single-task performed in the same session. The decrease was significant for both SSST and GPT for all comparisons (*p* < 0.0001; before, during and after; paired two-sample *t*-test). Similarly, the difference in performance between dual and single-task, expressed as dual-task costs, showed reduced dual-task performance.

**Table 2:** Dual-task (DT) performance: number of pegs inserted (GPT) and number of correct subtractions (SSST) within 30 s before (PRE), during (DUR) and 30 min after (POST), unilateral (Ul), bilateral (Bl) and sham tDCS of left (AL) and right (AR) DLPFC.

	BAL	BAR	UAL	UAR	Sham
SSSTDTPRE	8.13 ± 1.9	8.13 ± 2.1	8.22 ± 1.8	8.13 ± 1.3	8.31 ± 1.9
SSSTDTDUR	9.63 ± 1.9	8.36 ± 1.4	8.59 ± 1.6	8.5 ± 1.8	8.45 ± 1.7
SSSTDTPOST	9.13 ± 1.5	8.81 ± 1.6	8.72 ± 1.9	8.59 ± 2	8.86 ± 2
GPTDTPRE	12.09 ± 1.8	12.18 ± 1.9	11.9 ± 1.8	12 ± 1.8	12.18 ± 1.7
GPTDTDUR	13.4 ± 1.5	12.59 ± 1.9	12 ± 2.1	12.09 ± 1.8	12 ± 1.8
GPTDTPOST	12.59 ± 1.8	12.54 ± 2	12.04 ± 1.7	12.31 ± 1.8	12.27 ± 1.9

*Data are expressed as mean ± SD*.

### Effects of tDCS Montage on the Dual-Task Costs to Manual Dexterity (GPT Performance) and Serial Seven Subtraction Task (SSST)

We explored whether tDCS of the DLPFC cortex could improve dual-task performance and whether this effect depends on the stimulation polarity and orientation, i.e., montage. Thus, we used a mixed linear model (as described above) with dual-task costs as outcome (dependent). As shown in Table 3, these analyses show that dual-task costs were not significantly different among montages (sessions) before tDCS. In contrast, during tDCS bilateral tDCS with anode left, exerted a significant reduction in both SSST and GTP dual-task costs. The effect was more pronounced for SSST than for the manual dexterity task. Thirty minutes after tDCS the number of numbers subtracted, and pegs inserted were not significantly different among montages except for the number of pegs (manual dexterity) after UAL tDCS.

**Table 3:** Results of the generalized linear mixed model using montage to examine the SSST and GPT performance, before, during and after tDCS.

	SSTCost Before				SSTCost During				SSTCost After			
	Estimate	*p*	95% Conf Int		Estimate	*p*	95% Conf Int		Estimate	*p*	95% Conf Int	
BAL	0.018182	0.477	−0.032392	0.068756	−0.126841	0.001	−0.184939	−0.068743	−0.026259	0.362	−0.083200	0.030682
BAR	0.004509	0.860	−0.046065	0.055083	−0.027555	0.348	−0.085653	0.030544	−0.005023	0.861	−0.061964	0.051919
UAL	0.018395	0.471	−0.032179	0.068970	−0.020832	0.478	−0.078930	0.037267	0.006086	0.832	−0.050855	0.063028
UAR	0.01255	0.623	−0.038024	0.063124	−0.025382	0.387	−0.083480	0.032717	0.024264	0.399	−0.032678	0.081205
**GPTCost Before**				**GPTCost During**				**GPTCost After**			
	**Estimate**	***p***	**95% Conf Int**		**Estimate**	***p***	**95% Conf Int**		**Estimate**	***p***	**95% Conf Int**	
BAL	0.011586	0.594	−0.031504	0.054677	−0.056991	0.020	−0.104686	−0.009296	−0.011705	0.618	−0.058150	0.034741
BAR	0.027536	0.207	−0.015554	0.070627	−0.024768	0.305	−0.072463	0.022927	0.008491	0.717	−0.037954	0.054936
UAL	0.011936	0.583	−0.031154	0.055027	0.000109	0.996	−0.047586	0.047804	0.057595	0.016	0.011150	0.104041
UAR	0.0097	0.656	−0.033390	0.052790	−0.005432	0.821	−0.053127	0.042263	0.029573	0.209	−0.016873	0.076018

*Estimates refer to differences from Sham*.

## Discussion

This study was performed to investigate the “on-line” effects of tDCS of the DLPFC on dual-task performance in healthy aging. Our results extend earlier findings (Toosizadeh et al., 2016) by showing that performing a dual-task, one of which involves complex manipulative hand manual dexterity task, negatively affects the performance on both tasks. The results show that tDCS did not influence performance on any of the tasks when carried out in isolation. Bilateral tDCS, on the other hand, improved dual-task performance, but only with the anode positioned over the left DLPFC. The same montage was more effective in improving demanding cognitive performance than the manual dexterity task.

Most of the previous studies that examined changes in performance from single- to dual-task in older adults combined postural control while standing, or walking in combination with a cognitive task (e.g., Ruffieux et al., 2015). In this study dual-task performance was examined in a sitting position, combining a hand dexterity task and a mental arithmetic cognitive task. The number of pegs inserted within 30 s changed from an average of 14.4 during single-task to 12.1 during dual-task conditions, while serial subtraction decreased from 10.2 to 8.2. Thus, even while being seated performing two tasks simultaneously significantly deteriorated the performance on each task. Although not directly comparable, as some of the previous studies examined walking and standing while performing a cognitive task, the magnitude of dual-task costs at baseline (i.e., before real or sham tDCS) in this study are in line with previously reported ones (Hausdorff et al., 2008; Manor et al., 2016). This further confirms that the performance of healthy older adults deteriorates under dual-task conditions involving demanding cognitive tasks. The results of this study further expand the array of motor and cognitive tasks that incur dual-task cost in older adults.

DTC was marginally higher for GPT. Nevertheless, although some subjects on average had worse performance on one task while others had worse performance on the other task, there were no significant differences in DTC between two tasks. Previous studies that examined task performance under dual-task conditions showed that the performance depends on the priority given to each of the tasks (Yogev-Seligmann et al., 2010). Some earlier studies did not give specific instructions regarding task prioritization, while in this study subjects were specifically instructed and repeatedly reminded not to priorities any task. Also, most of the previous studies examined gait or postural task, in which a “posture-first” strategy is consciously or unconsciously employed to minimize the possibility of falling. In this study, the subjects were seated, thus excluding the “pressure” to prioritize motor task.

Previous studies that examined the effects of tDCS on DTC in older adults showed that tDCS does not alter single-task performance (Zhou et al., 2014, 2015; Manor et al., 2016). Similarly, in this study tDCS, when applied concomitantly with the task (i.e., in an on-line regimen) did not alter the performance of either of the single tasks. The only other study that used GPT with tDCS applied the stimulation over motor cortex and showed that tDCS combined with practice did not affect performance on the GPT but prolonged the retention of improved GPT performance (Parikh and Cole, 2014). Other studies that applied tDCS over M1 also showed improved performance of a single dexterity demanding task (Hummel et al., 2010; Pavlova et al., 2014; Parikh and Cole, 2015). In contrast, it has been reported that two mA tDCS over motor cortex did not have effects on the performance and practice of GPT (Fagerlund et al., 2015). The overall absence of effects of tDCS, unilateral or bilateral, anodal or cathodal, on a single task, and particularly on GPT, in this study may suggest that the functional integrity of the underlying neural network was preserved or performing at its maximum and could not be further augmented by the stimulation.

Unlike the effects on single-task, tDCS induced significant effects in dual-task condition as evidenced by changes in DTC. However, the effects were present only with bilateral DLPFC stimulation and were present only with an anodal left-cathodal right montage. Contrary, previous studies showed improved dual-task performance with unilateral tDCS of the left DLPFC (Zhou et al., 2014, 2015). In two studies the effects were more prominent on gait and postural motor tasks than on cognitive tasks (Zhou et al., 2014, 2015), while one reported reduced dual-task costs for both standing and walking, and serial subtraction (Manor et al., 2016). Contrary to these studies, unilateral tDCS had no effects on dual-task costs, while bilateral stimulation had limited effects on dexterity-motor dual-task costs. It seems unlikely that this difference may be related to the intensity and duration of the stimulation. We applied 1.5 mA for 30 min while other studies used either two mA (Zhou et al., 2015; Manor et al., 2016) or 1.5 mA (Zhou et al., 2014) for 20 min. Furthermore, the difference is unlikely to have been caused by electrode placement. In this study, similar to previous studies, for unilateral tDCS the active electrode (UAR or UAL) was placed over the left DLPFC area (F3) while the return electrode was placed over the contralateral supraorbital region, most likely giving a comparable current distribution and cortical activation. The potential difference between tDCS effects may be related to a different motor task examined in this study, i.e., GPT. It has been shown, that the performance on GPT strongly correlates with several aspects of neurodegeneration like the cognitive impairment (Bezdicek et al., 2014) and dopaminergic nigrostriatal denervation in Parkinson’s disease (Bohnen et al., 2007), as well as with white matter lesions in normal aging (Nyquist et al., 2015). Thus, it may be that the manipulative manual dexterity task like GPT, which operates across different sensory and motor domains, may require stronger or more widespread cortical activation, like the one induced by bilateral tDCS in this study. Finally, unlike in previous studies, tDCS in this study was more effective on cognitive than on dexterity task performance. Namely, the SSST dual-cost improved significantly changing from 8.1 correct subtractions before tDCS to 9.6 correct subtractions during tDCS. Unlike, the SSST, the GPT went from 12.1 inserted pegs to 13.4 during tDCS. It has been proposed that humans are limited in their capacity to reliably perform more than one task, i.e., to multitask, mostly due to the inability of cortical networks shared between two tasks to process both at the same time (i.e., processing bottlenecks; Pashler and O’Brien, 1993). This would cause serial rather than parallel processing, and diminish the performance of either of the two tasks or both (Ruthruff et al., 2001; Sigman and Dehaene, 2006). It has been consistently reported by fMRI studies that performance of both single- and dual-tasks in older adults requires heightened brain activation (Park and McDonough, 2013). During dual-task calculation, the most involved areas include the bilateral precentral gyri, the left medial frontal gyrus, bilateral lingual gyri, and right inferior and middle occipital gyrus (Papegaaij et al., 2017). It seems as if dual-task coordination does not depend exclusively on one prefrontal area but rather involves the interplay of various specialized information-processing sub-systems. In this study only, bilateral anodal stimulation significantly reduced dual-task costs, i.e., improved cognitive performance. It has been reported earlier that anodal tDCS co-administered with cognitive tasks can significantly enhance working memory performance (Katsoulaki et al., 2017). It is generally assumed that tDCS modulates cortical excitability in a polarity-dependent fashion so that anodal stimulation increases intracortical facilitation and diminish intracortical inhibition, while cathodal tDCS has the reverse effect (Nitsche et al., 2008). The finding that both unilateral stimulation montages (UAR or UAL) failed to enhance or even diminish the dual-task performance may suggest that stimulating DLPFC may not be optimal when performing these two tasks. This is consistent with the notion that dual-task coordination is not exclusively dependent on prefrontal areas but rather involves the interplay of various inter-connected networks. Nevertheless, since bilateral anodal tDCS improved the cognitive dual-task costs suggests that DLPFC, at least in part, is involved in dual-tasking, while other areas, as suggested by imaging studies, may be equally involved. Along these lines, the significant effect of bilateral tDCS may be explained by its overall effects on complex network modulations involving interhemispheric interactions and areas related to motor control in the dorsal posterior cingulate cortex (Lindenberg et al., 2013), rather than by simple concurrent anodal-cathodal effects. Furthermore, since tDCS reduced the cost of performing a cognitive task on motor dexterity, and that at the same time the cost induced by a motor task on mental arithmetic did not change significantly suggests that tDCS has probably augmented efficient utilization of involved extensive networks rather than causing the reallocation of available resources between the tasks. Finally, although the general trend for dual-task performance improvement remained, only BAR tDCS induced significant changes in performance 30 min after the stimulation. This suggests that single tDCS may have limited effects and that lasting improvements may require multiple sessions and different regimen like off-line or combined on-line, off-line stimulation.

This is the first study that examined “on-line” tDCS on dual-tasking involving manual dexterity in older adults. All previous studies used tDCS “preconditioning,” i.e., dual-tasking was performed after tDCS session. It should be noted though that in this study tDCS was started before testing but continued during the single- and dual-tasks. It can be argued that at least in part DLPFC was preconditioned. While we cannot entirely rule out the relevance of preconditioning, we believe that the ongoing effects of tDCS while performing the task probably exerted stronger potentiating effects than “preconditioning.” Along these lines, a recent study showed that concurrent application of tDCS with a motor task might exert larger sensorimotor cortex activation than the sequential application (Besson et al., 2019).

## Conclusion

Single bilateral tDCS session of DLPFC improves the performance of demanding cognitive task while dual tasking with demanding dexterity task in older individuals. Further studies are needed to elucidate whether these effects could be extended, particularly with repeated stimulation. This could strengthen the argument to explore further the use of tDCS to improve motor performance and cognitive functioning in older adults.

## Ethics Statement

All subjects gave written informed consent to participate in the experiment in accordance with the Declaration of Helsinki. The Al Ain Medical District Human Research Ethics Committee approved the study (Protocol No. 14/57).

## Author Contributions

ML designed the study, performed experiments, analyzed the data and wrote a manuscript draft. JO performed the experiments and helped in data analysis. SF helped in data interpretation and manuscript writing. JB helped with data analysis and manuscript proofreading. MS helped in study design, data interpretation, and manuscript proofreading. NN helped in study design, statistical analysis, and manuscript proofreading.

## Conflict of Interest Statement

The authors declare that the research was conducted in the absence of any commercial or financial relationships that could be construed as a potential conflict of interest.
